# Particulate matter and emergency visits for asthma: a time-series study of their association in the presence and absence of wildfire smoke in Reno, Nevada, 2013–2018

**DOI:** 10.1186/s12940-020-00646-2

**Published:** 2020-08-27

**Authors:** Daniel Kiser, William J. Metcalf, Gai Elhanan, Brendan Schnieder, Karen Schlauch, Andrew Joros, Craig Petersen, Joseph Grzymski

**Affiliations:** 1 Renown Institute for Health Innovation, Reno, Nevada USA; 2 Division of Earth and Ecosystem Sciences, Desert Research Institute, Postal – 2215 Raggio Pkwy, Reno, Nevada NV 89512-1095 USA; 3 Washoe County Health District Air Quality Management Division, Reno, Nevada USA

**Keywords:** Asthma, Wildfires, Particulate matter, Smoke, Emergency department, Urgent care, Reno, Nevada, Generalized additive model, Interaction

## Abstract

**Background:**

Health risks due to particulate matter (PM) from wildfires may differ from risk due to PM from other sources. In places frequently subjected to wildfire smoke, such as Reno, Nevada, it is critical to determine whether wildfire PM poses unique risks. Our goal was to quantify the difference in the association of adverse asthma events with PM on days when wildfire smoke was present versus days when wildfire smoke was not present.

**Methods:**

We obtained counts of visits for asthma at emergency departments and urgent care centers from a large regional healthcare system in Reno for the years 2013–2018. We also obtained dates when wildfire smoke was present from the Washoe County Health District Air Quality Management Division. We then examined whether the presence of wildfire smoke modified the association of PM_2.5_, PM_10–2.5_, and PM_10_ with asthma visits using generalized additive models. We improved on previous studies by excluding wildfire-smoke days where the PM concentration exceeded the maximum PM concentration on other days, thus accounting for possible nonlinearity in the association between PM concentration and asthma visits.

**Results:**

Air quality was affected by wildfire smoke on 188 days between 2013 and 2018. We found that the presence of wildfire smoke increased the association of a 5 μg/m^3^ increase in daily and three-day averages of PM_2.5_ with asthma visits by 6.1% (95% confidence interval (CI): 2.1–10.3%) and 6.8% (CI: 1.2–12.7%), respectively. Similarly, the presence of wildfire smoke increased the association of a 5 μg/m^3^ increase in daily and three-day averages of PM_10_ with asthma visits by 5.5% (CI: 2.5–8.6%) and 7.2% (CI: 2.6–12.0%), respectively. We did not observe any significant increases in association for PM_10–2.5_ or for seven-day averages of PM_2.5_ and PM_10_.

**Conclusions:**

Since we found significantly stronger associations of PM_2.5_ and PM_10_ with asthma visits when wildfire smoke was present, our results suggest that wildfire PM is more hazardous than non-wildfire PM for patients with asthma.

## Introduction

Studies have shown that particulate matter (PM) from wildfires can have an adverse effect on the health of patients with asthma [1–5]. However, it is not yet clear whether wildfire PM is more or less harmful for health conditions like asthma than PM from other sources, such as transportation and industry [6, 7]. Differences in the effects of wildfire and non-wildfire PM seem likely, since several in vitro studies [8–10] and at least one study using live mice [11] have reported differences in biological and chemical responses to wildfire and non-wildfire PM. There have also been several population health studies that have explicitly compared the effects of wildfire and non-wildfire PM on patients with asthma [12–14]. While these studies generally found a stronger association of adverse asthma events with wildfire PM than with non-wildfire PM, the differences were often not statistically significant. Furthermore, since concentrations of PM tend to be much higher during wildfire events, it is possible that the differences between wildfire and non-wildfire PM found in these studies were due to nonlinearity in the association between PM and adverse asthma events.

An accurate understanding of the impacts of wildfire smoke on population health is critical if forest management policies are to effectively mitigate those impacts. The need to improve such policies is especially urgent for population health in places like Reno, Nevada, where wildfire smoke events occur regularly during the warm season. These events are currently expected to become more frequent in Reno, as the mean amount of area burned each year in the region immediately west of Reno is predicted to increase by 78% from the period 1996–2005 to the period 2046–2055, due to climate change [15].

Because of its frequent smoke exposures, Reno is ideal for studying the health effects of wildfire smoke. However, it is also suitable because it is home to a single regional healthcare system, Renown Health, that is estimated to receive 68.4% of the acute care cases in northern Nevada (Alan Herak, Director of Decision Support, Renown Health, personal communication, May 14, 2019; estimate based on state-wide health billing data [16]). Because of its dominant market share, Renown’s electronic health records (EHR) offer a comprehensive view of acute health problems resulting from wildfires occurring near Reno. Reno and neighboring Sparks (which we refer to collectively as Reno) have a combined population of approximately 355,000 [17] and are located in an intermountain desert valley at about 4500 ft in elevation.

Only two previous studies have examined the association of PM with asthma visits in Reno. In the first, Yang et al. [18] found no association between PM_10_ (PM between 0 and 10 μm in diameter) and emergency department (ED) visits. In the second, Rosenquist et al. [19] found associations between PM_2.5_ (PM less than 2.5 μm in diameter, or fine PM) and any type of asthma visit—such as office visits, ED visits, urgent care visits, or hospitalizations—with differences in association for allergic and nonallergic asthma patients. Neither Rosenquist et al. nor Yang et al. examined how the association of PM with asthma visits may be modified during wildfires. However, Rosenquist et al. speculated that they may have seen elevated risk associated with PM_2.5_ during the warm season due to differences in the composition of wildfire PM_2.5_ compared to non-wildfire PM_2.5_.

Our goal in this study was to estimate the extent to which the presence of wildfire smoke changes the association between PM and daily counts of asthma visits to EDs and UC centers in Reno, and hence to investigate whether PM from wildfires is more or less hazardous for asthma patients than PM from other sources.

## Methods

### Data

**Fig. 1:**
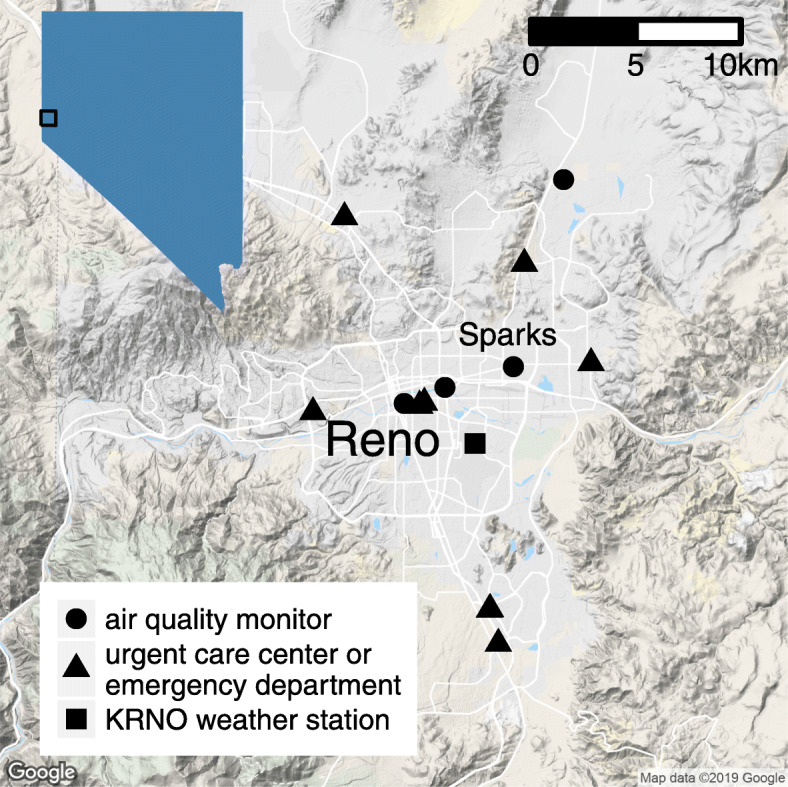
Map of the Reno-Sparks area showing the locations of air quality monitors, urgent care centers, emergency departments, and the KRNO weather station

We obtained daily measurements of 24-h average PM_2.5_, 24-h average PM_10–2.5_ (PM between 2.5 and 10 μm in diameter, or coarse PM), and 24-h average PM_10_ for the years 2013 through 2018 from four air quality monitors in the Reno area (Fig. 1). We used only the measurements for each PM fraction that were reported under local conditions to ensure consistency across measurements. Population exposure to each fraction of PM was estimated using a weighted average of the monitors that were active on each day. As weights, we used the counts of Renown patients whose last known address was within a 5 km radius of each monitor (with the exception of the Galletti monitor, where we used a radius of 2 km due to a nearby sandlot that affected the local air quality). Only one of the four monitors was active for the entire study period for all three PM fractions.

We also obtained qualifier data, which is an hour by hour list of events that may have affected pollutant measurements, from the Washoe County Health District Air Quality Management Division (WCHD-AQMD). These events include monitor malfunctions, maintenance, or calibration, as well as the presence of smoke from a wildfire or a prescribed burn. This data is submitted to the Environmental Protection Agency (EPA) quarterly and is subject to EPA audits. However, while every agency is required to record events such as monitor maintenance and calibration, recording smoke events is voluntary, and thus data on smoke events is not necessarily available from every air monitoring agency. WCHD-AQMD began recording smoke events consistently at the beginning of 2013, and these events were determined primarily by the following criteria:

1. Elevated levels of organic carbon in speciated PM_2.5_
2. High concentrations of PM_2.5_ for the time of year (greater than 12 μg/m^3^ during the warm season was considered unusual)
3. High concentrations of PM_2.5_ compared to recent data (i.e. a spike)
4. Deviations of PM_2.5_ from its typical diurnal pattern in the warm season

Other criteria that were used but which carried less weight than the above criteria were concentrations of PM_10_, PM_10–2.5_, nitrogen oxides, and ozone; whether nitrogen oxides and ozone deviated from their diurnal patterns; visibility at the Reno-Tahoe Airport; forecasts by the National Weather Service; the ratio of PM_2.5_ to PM_10_, with a ratio greater than 0.5 being considered indicative of wildfire smoke; the ratio of carbon monoxide to PM_2.5_; satellite images; and HYSPLIT (Hybrid Single Particle Lagrangian Integrated Trajectory) models.

For our analysis, we did not make a distinction between smoke from a wildfire and smoke from a prescribed burn, and we considered the air quality on any given day to be affected by wildfire smoke if any hour of that day was affected, as indicated by the qualifier data from the Reno3 air quality monitor (which is located in downtown Reno). We henceforth refer to days where the air quality was affected by wildfire smoke as “wildfire days” and days where the air quality was not affected by wildfire smoke as “non-wildfire days”.

Hourly temperature and humidity data were obtained from the KRNO weather station located at the Reno-Tahoe Airport (Fig. 1). We calculated the daily minimum, mean, and maximum values for temperature, and the daily mean for relative humidity (RH). Visits for asthma were defined as hospital or office visit encounters with an associated diagnosis code matching ICD-10 codes J45.20-J45.998 and/or ICD-9 codes 493.00–493.92. Daily counts of visits for the years 2013–2018 were obtained from Renown’s EHR for two EDs and seven UC centers (Fig. 1). We had full access to Renown’s EHR for the time period under investigation.

### Analyses

For modeling, we used a generalized additive model (GAM) from the Negative Binomial family, a distribution family which allows for overdispersed Poisson outcomes. To construct our models, we first created a base model that had the following form:

$$ \log \left(E(visits)\right)=\alpha +\gamma DOW+\delta holidays+ cr(time)+\zeta temp+\eta AR $$

where visits represents the daily count of asthma visits, *DOW* represents six separate indicator variables for days of the week, *holidays* represents 17 separate indicator variables for selected holidays, *cr*(*time*) represents a cubic regression spline of time (in days), *temp* represents a rolling seven-day average of daily mean temperature, and *AR* represents autoregressive terms introduced to control for autocorrelation. After examining autocorrelation of the base model residuals using autocorrelation function (ACF) and partial ACF plots, we added autoregressive terms for lags 1–10 of visits. Degrees of freedom for *cr*(*time*) were not selected a priori, since the R package used for building the GAMs, **mgcv**, uses a penalized regression spline approach that automatically determines the degree of smoothness [20].

In order to examine both immediate and delayed associations between asthma visits and PM, we constructed variables for each PM fraction (PM_2.5_, PM_10–2.5_, and PM_10_) representing immediate exposure (lag 0), short-term cumulative exposure (rolling average of lags 0–2), and longer-term cumulative exposure (rolling average of lags 0–6). We similarly constructed indicator variables for days affected by wildfire smoke: an indicator for at least one wildfire day (lag 0), an indicator for at least three wildfire days in a row (lags 0–2), and an indicator for at least seven wildfire days in a row (lags 0–6). The last two wildfire indicators had the value “1” when all of the included lags were wildfire days, but “0” only when none of the included lags were wildfire days. If the lags were a mix of wildfire days and non-wildfire days, we excluded that observation from the analysis whenever that indicator was used. In our models described below, we always use the indicator variable for wildfires whose lags correspond to the lags used to construct the PM variable (lags 0, 0–2, and 0–6). Finally, we also constructed variables indicating the daily activity status of each air quality monitor, to account for differences in air quality at each monitoring site. For models that included lag 0–2 and lag 0–6 averages of PM, these variables indicated whether a particular monitor was active at any lag.

Because of the possibility of a nonlinear association between PM and asthma visits (such as a stronger association at higher concentrations of PM than at lower concentrations), we excluded wildfire days if the concentration of PM_2.5_ on those days exceeded the maximum PM_2.5_ concentration observed on non-wildfire days. This was done separately for each set of lags, as a high value of PM_2.5_ on one day would not necessarily cause a rolling average of PM_2.5_ concentrations on wildfire days to exceed the maximum of the rolling average on non-wildfire days. We only excluded days on the basis of PM_2.5_, and not PM_10–2.5_ or PM_10_, since the maximum concentration of PM_10–2.5_ was higher on non-wildfire days than on wildfire days.

For our analyses, the values of PM_2.5_, PM_10–2.5_, and PM_10_ were scaled so that a unit change is equivalent to 5 μg/m^3^, and we defined statistical significance as *p* < 0.05. In order to determine whether PM is more hazardous on wildfire days than on non-wildfire days, we modeled an interaction between PM and wildfires as follows:

1 $$ \boldsymbol{\log}\left(\boldsymbol{E}\left(\boldsymbol{visits}\right)\right)=\boldsymbol{\alpha} +\boldsymbol{\gamma} \boldsymbol{DOW}+\boldsymbol{\delta} \boldsymbol{holidays}+\boldsymbol{cr}\left(\boldsymbol{time}\right)+\boldsymbol{\zeta} \boldsymbol{temp}+\boldsymbol{\eta} \boldsymbol{AR}+{\beta}_1 PM+{\beta}_2\left( PM\times WF\right)+{\beta}_3 WF+\theta MO{N}_{PM} $$

In this equation, terms in bold belong to the base model. *PM* represents any of the three fractions of PM (PM_2.5_, PM_10–2.5_, or PM_10_) from any of the three lag sets (lags 0, 0–2, or 0–6), while *WF* represents the corresponding wildfire indicator from the same lag set. *MON*_*PM*_ represents the set of indicator variables for the activity status of each monitor. We constructed nine different models of this form, one for each combination of PM fraction and lag sets.

In these models, which we refer to as the “single-fraction models”, the rate ratio for the change in the number of asthma visits due to a 5 μg/m^3^ change in PM is given by: $\exp \left(\hat{\beta_1}+\hat{\beta_2} WF\right)$, where $\exp \left(\hat{\beta_1}\right)$ is the rate ratio when wildfire smoke is not present (i.e., *WF* = 0) and $\exp \left(\hat{\beta_1}+\hat{\beta_2}\right)$ is the rate ratio when wildfire smoke is present (i.e, *WF* = 1). $\exp \left(\hat{\beta_2}\right)$ quantifies the multiplicative change in the rate ratio when wildfire smoke is present, and allows us to construct confidence intervals for this change. We also report this change as a percent: $\left(\exp \left(\hat{\beta_2}\right)-1\right)\times 100$. To estimate the variance of *β*_1_ + *β*_2_, we used the standard formula for the variance of a linear combination of random variables, similar to Deflorio-Barker et al. [12].

We also created an additional model, which we refer to as the “two-fraction model”, that allowed us to predict the increase in asthma visits due to the presence of wildfire smoke for any given levels of PM_2.5_ and PM_10–2.5_. This model included terms for both lag-0 PM_2.5_ and PM_10–2.5_ and their respective interactions with the wildfire indicator:

2 $$ \boldsymbol{\log}\left(\boldsymbol{E}\left(\boldsymbol{visits}\right)\right)=\boldsymbol{\alpha} +\boldsymbol{\gamma} \boldsymbol{DOW}+\boldsymbol{\delta} \boldsymbol{holidays}+\boldsymbol{cr}\left(\boldsymbol{time}\right)+\boldsymbol{\zeta} \boldsymbol{temp}+\boldsymbol{\eta} \boldsymbol{AR}+{\beta}_1P{M}_{2.5}+{\beta}_2\left(P{M}_{2.5}\times WF\right)+{\beta}_3P{M}_{10-2.5}+{\beta}_4\left(P{M}_{10-2.5}\times WF\right)+{\beta}_5 WF+\theta MO{N}_{PM2.5}+\lambda MO{N}_{PM10-2.5} $$

In contrast to equation (1), the *PM* and *WF* terms in equation (2) indicate only lag 0 constructions, while *MON*_*PM*2.5_ and *MON*_*PM*10 − 2.5_ represent sets of indicators for monitors actively measuring PM_2.5_ and PM_10–2.5_, respectively. The estimated percent increase in asthma visits due to the presence of wildfire smoke at given levels of PM_2.5_ and PM_10–2.5_ (scaled to 5 μg/m^3^) was calculated as:

3 $$ \left(\exp \left(\hat{\beta_2}P{M}_{2.5}+\hat{\beta_4}P{M}_{10-2.5}+\hat{\beta_5}\right)-1\right)\times 100 $$

The variance of the expression *β*_2_*PM*_2.5_ + *β*_4_*PM*_10 − 2.5_ + *β*_5_, which was used to calculate *p*-values, was calculated using the standard formula for the variance of a linear combination of random variables, as follows:

$$ Var\left({\beta}_2P{M}_{2.5}+{\beta}_4P{M}_{10-2.5}+{\beta}_5\right)=P{M}_{2.5}^2 Var\left(\hat{\beta_2}\right)+P{M}_{10-2.5}^2 Var\left(\hat{\beta_4}\right)+ Var\left(\hat{\beta_5}\right)+2P{M}_{2.5}P{M}_{10-2.5} Cov\left(\hat{\beta_2},\hat{\beta_4}\right)+2P{M}_{2.5} Cov\left(\hat{\beta_2},\hat{\beta_5}\right)+2P{M}_{10-2.5} Cov\left(\hat{\beta_3},\hat{\beta_5}\right) $$

As suggested by the above formula, the level of significance of the estimates depends on the values of *PM*_2.5_ and *PM*_10 − 2.5_.

To test the stability of the models, we repeated the analysis using different controls for meteorological factors. Specifically, we replaced the rolling seven-day average of daily mean temperature with a rolling seven-day average of either daily maximum temperature or daily minimum temperature. We also repeated the analysis using a rolling seven-day average of daily mean RH in addition to our control for daily mean temperature. We then compared the results of the sensitivity analyses to that of the primary analysis. We used R version 3.6.0 and the R packages **mgcv 1.8–28** [20], **mgcv.helper 0.1.8** [21], and **TSA 1.2** [22] for all of our statistical modeling, and we used SAS 9.4 for extracting data from Renown’s EHR. Work on this study was Institutional Review Board (IRB) exempt by the University of Nevada, Reno (UNR) IRB as part of a larger body of work, “Interoperability, operational efficiency and quality of care improvements through health data analysis.”

## Results

**Fig. 2:**
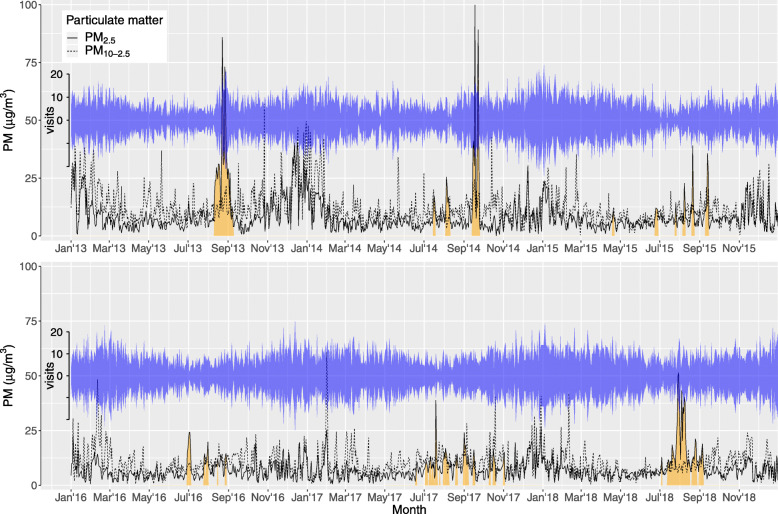
Time series of particulate matter and asthma visits from January 1, 2013, to December 31, 2018 in Reno, Nevada. Daily values of PM_2.5_ and PM_10–2.5_ are weighted averages across all air quality monitors. The width of the blue ribbon indicates the number of asthma visits, and the orange-shaded regions below the time series for PM_2.5_ indicate days where the air quality was determined by WCHD-AQMD to be affected by wildfire smoke

Air quality was affected by wildfire smoke on 188 days between 2013 and 2018 (Fig. 2). Of these days, there were 125 days that were preceded by at least two other wildfire days, and 68 days that were preceded by at least six other wildfire days. Although we defined wildfire days as days where the air quality was affected by wildfire smoke for at least one hour, air quality on wildfire days was generally affected by smoke for most of the day, with 182 days affected by smoke for more than 20 h and only 6 days affected by smoke for less than 20 h (Figure S1). Inspection of the data revealed that small decreases in the number of hours recorded as affected by wildfire smoke on any given day were often caused by brief periods of monitor malfunction or maintenance. We found that the maximum levels of PM_2.5_ for non-wildfire days were 40.4 μg/m^3^ (lag 0), 36.8 μg/m^3^ (rolling average of lags 0–2), and 35.0 μg/m^3^ (rolling average of lags 0–6). After excluding wildfire days where PM_2.5_ exceeded these maximums, we retained 175 solitary wildfire days, 107 wildfire days where the previous 2 days were also wildfire days, and 50 wildfire days where the previous 6 days were also wildfire days. The most severe wildfire smoke events, with the highest PM_2.5_ levels and the longest duration, occurred in August–September of 2013, September of 2014, and July–September of 2018 (Fig. 2).

As expected, concentrations of PM_10_ were highest on days affected by wildfire smoke, with mean concentrations of PM_10_ increasing from 17.9 μg/m^3^ on non-wildfire days to 31.2 μg/m^3^ on wildfire days (Table 1). However, most of that increase was due to PM_2.5_, for which mean concentrations increased from 7.1 μg/m^3^ on non-wildfire days to 19.4 μg/m^3^ on wildfire days, while mean concentrations of PM_10–2.5_ only increased from 10.9 μg/m^3^ to 11.6 μg/m^3^. In fact, maximum concentrations of PM_10–2.5_ were higher on non-wildfire days than on wildfire days. The variation in PM_10–2.5_ was also higher on non-wildfire days than on wildfire days, with the standard deviation (SD) increasing from 4.8 to 7.1, in contrast to the SDs for PM_2.5_ and PM_10_, which decrease from 14.8 to 4.8 and from 16.5 to 10.6, respectively. When we examine the Pearson correlations among the different size fractions of PM (Table 2), we note that concentrations of PM_10_ were correlated with both PM_2.5_ and PM_10–2.5_ on non-wildfire days (0.87 and 0.94, respectively), but that during wildfires PM_10_ was more correlated with PM_2.5_, with a correlation of 0.96, than with PM_10–2.5_, with a correlation of 0.46.

**Table 1:** Daily mean, range, and standard deviation for asthma visits, PM, and weather factors

	All days		Non-wildfire days		Wildfire days	
Variable	mean (range)	SD	mean (range)	SD	mean (range)	SD
Asthma visits	8.6 (0–25)	3.9	8.6 (0–25)	3.8	8.2 (1–21)	3.9
24-h PM 2.5 (μg/m^3^)	8.1 (0.1–99.9)	7.2	7.1 (0.1–40.4)	4.8	19.4 (3.9–99.9)	14.8
24-h PM 10–2.5 (μg/m^3^)	11.0 (0.4–60.8)	6.9	10.9 (0.4–60.8)	7.1	11.6 (3.2–40.3)	4.8
24-h PM 10 (μg/m^3^)	19.0 (2.0–118.3)	11.9	17.9 (2.0–86.3)	10.6	31.2 (10.3–118.3)	16.5
Mean temperature (°C)	13.3 (−12.2–33.1)	9.1	12.2 (−12.2–33.1)	8.7	24.4 (10.9–31.7)	4.0
Mean relative humidity (%)	43.0 (12.3–92.3)	17.1	44.3 (12.3–92.3)	17.1	29.2 (15.3–88.8)	9.7

*SD* standard deviation

**Table 2:** Pearson correlations between daily averages of PM and weather factors

	All days				Non-wildfire days				Wildfire days			
Variable	PM_**10–2.5**_	PM_**10**_	Temp	RH	PM_**10–2.5**_	PM_**10**_	Temp	RH	PM_**10–2.5**_	PM_**10**_	Temp	RH
**PM**_**2.5**_	0.45	0.86	0.00	0.05	0.67	0.87	−0.30	0.28	0.19	0.96	−0.05	0.03
**PM**_**10–2.5**_		0.84	−0.15	0.01		0.94	−0.17	0.02		0.46	−0.30	0.05
**PM**_**10**_			−0.07	0.03			−0.23	0.13			−0.14	0.04
**Temp**				−0.78				−0.78				−0.44

*RH* daily mean relative humidity, *Temp* daily mean temperature

There were 18,836 ED and UC center visits for asthma between 2013 and 2018. The average number of daily visits for asthma were slightly higher on non-wildfire days than on wildfire days (8.6 versus 8.2 visits, Table 1). This small decrease in daily visits on wildfire days can be attributed to a generally higher number of daily visits during the cold season than during the warm season (Fig. 2), when wildfires are likely to occur.

**Fig. 3:**
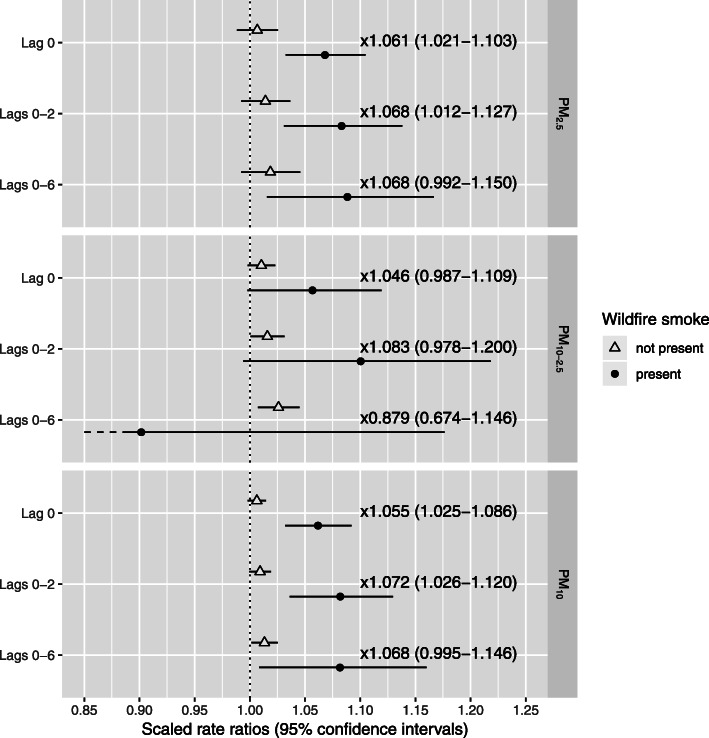
Rate ratios for the effect of PM on asthma visits to the ED and UC centers in the presence or absence of wildfire smoke. PM was scaled to 5 μg/m^3^ for all fractions and lags. Numeric labels between points are estimates of the change in the rate ratio when wildfire smoke is present compared to when it is not present, presented as a factor. This change can also be presented as a percent (i.e., the factor 1.028 is equivalent to an increase of 2.8%), which is how the changes are presented in the body of the paper. Rate ratios and the differences between them are presented in tabular form in Table S1

In the single-fraction models (see equation 1), we found that PM had a positive—though generally not statistically significant—association with asthma visits for all combinations of lags and PM fractions on non-wildfire days (Fig. 3). However, with the exception of lags 0–6 PM_10–2.5_, rate ratios were larger on wildfire days. This increase in rate ratio was significant for PM_2.5_ and PM_10_ at lag 0 and lags 0–2. The rate ratio for a 5 μg/m^3^ change in lag 0 PM_2.5_ increased from 1.007 (CI: 0.988–1.026) to 1.068 (CI: 1.033–1.105), an increase of 6.1% (CI: 2.1–10.3%), while the rate ratio for lags 0–2 PM_2.5_ increased from 1.014 (CI: 0.992–1.037) to 1.083 (CI: 1.031–1.138), an increase of 6.8% (CI: 1.2–12.7%). Similarly, the rate ratio for a 5 μg/m^3^ change in lag 0 PM_10_ increased from 1.006 (CI: 0.998–1.015) to 1.062 (CI: 1.032–1.092), an increase of 5.5% (CI: 2.5–8.6%), while the rate ratio for lags 0–2 PM_10_ increased from 1.009 (CI: 0.999–1.019) to 1.082 (CI: 1.036–1.130), an increase of 7.2% (CI: 2.6–12.0%). We did not observe any significant increases in association for PM_10–2.5_ or for lags 0–6 PM_2.5_ and PM_10_. Confidence intervals for PM_10–2.5_ on wildfire days were much wider than the confidence intervals for either PM_2.5_ or PM_10_ on wildfire days. It is important to emphasize that the magnitude of the changes in association described above varies with the unit scale used for PM, even though the statistical significance of the changes remains the same.

**Fig. 4:**
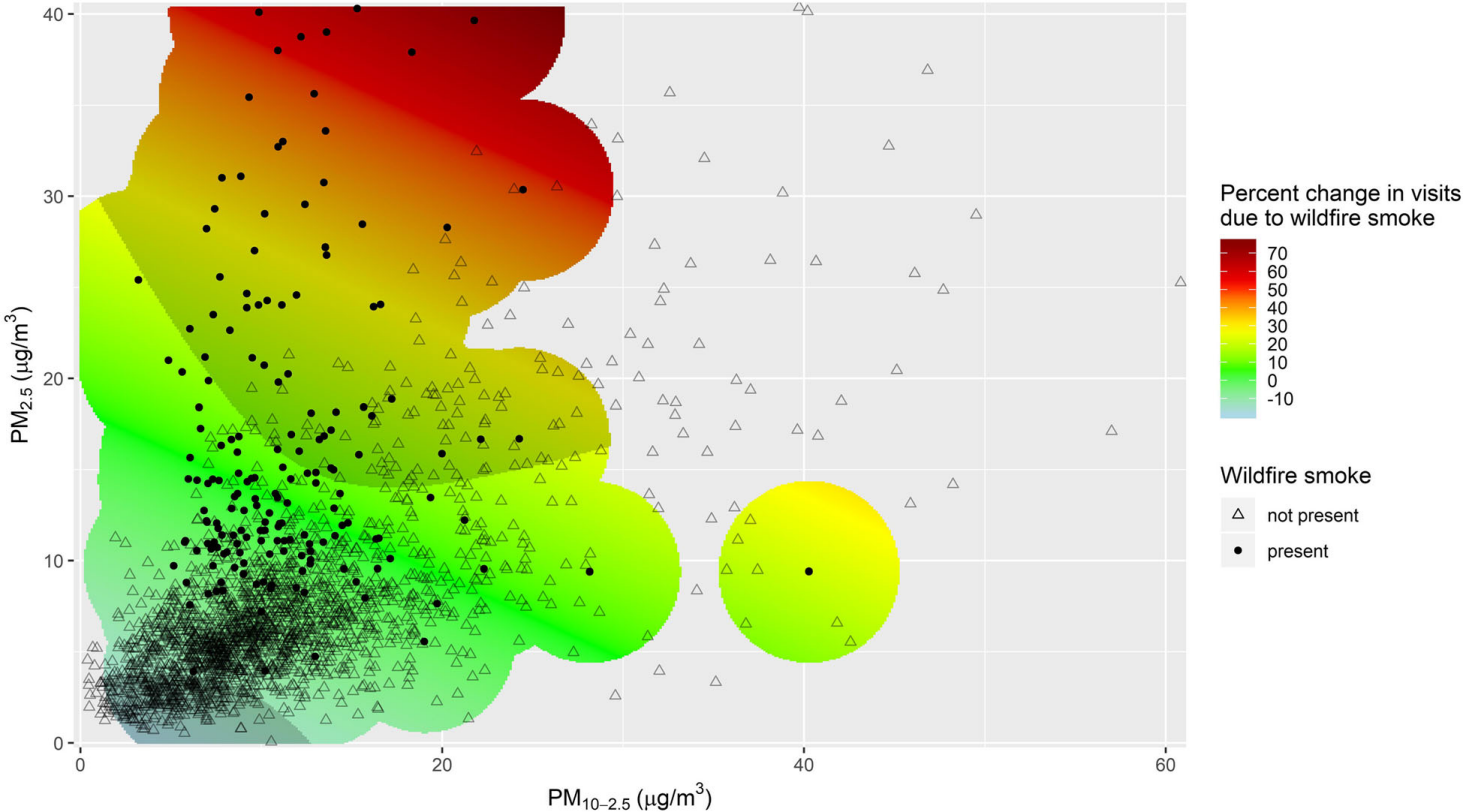
Percent increase in asthma visits due to wildfire smoke, at given levels of PM_2.5_ and PM_10–2.5_. Estimates were constrained to the regions within 5 μg/m^3^ of a wildfire data point to limit extrapolation. Shaded regions indicate estimates that were significant at the 0.05 level

The results of the two-fraction model (see equation 2) are visualized in Fig. 4. Instead of examining the association between PM and asthma visits and how it changes in the presence of wildfire smoke, as in Fig. 3, we here examine the association between wildfire smoke and asthma visits and how it changes at different levels of PM. The estimated percent change in asthma visits due to the presence of wildfire smoke is (exp(0.0747 × *PM*_2.5_ + 0.0341 × *PM*_10 − 2.5_ − 0.2259) − 1) × 100, where PM_2.5_ and PM_10–2.5_ are scaled to 5 μg/m^3^ (equation 3). Thus, we would expect the presence of wildfire smoke to increase the number of asthma visits by 19.1% when PM_2.5_ is at 20.0 μg/m^3^ and PM_10–2.5_ is at 15.0 μg/m^3^ (*p* = 0.00092). If both PM_2.5_ and PM_10–2.5_ increased by 5.0 μg/m^3^, to 25.0 and 20.0 μg/m^3^ respectively, we would then expect the presence of wildfire smoke to increase the number of asthma visits by 32.8% (*p* = 0.00022). In addition to limiting extrapolation of the model results, the overlaid scatterplot of observed values in Fig. 4 also illustrates the difference in the PM_2.5_/PM_10–2.5_ ratio on wildfire days and non-wildfire days. The mean PM_2.5_/PM_10–2.5_ ratio was 1.62 on wildfire days and 0.77 on non-wildfire days (after excluding wildfire days where PM_2.5_ exceeded the maximum PM_2.5_ of non-wildfire days).

The results of the sensitivity analyses (shown in the Supplementary materials) were nearly identical to the results of the primary analysis. However, estimates for non-wildfire days in our single-fraction models were slightly depressed when daily mean temperature was replaced with daily minimum temperature, and they were slightly inflated when daily mean temperature was replaced with daily maximum temperature (Tables S1, S2, S3 and Figs. 3, S2, S3). These changes in the estimates for non-wildfire days influenced the estimates for the change in association due to wildfires as well, such that estimates of change due to wildfires in the models controlling for daily minimum temperature were somewhat increased while the estimates of change due to wildfires in the models controlling for daily maximum temperature decreased. For example, the lag 0 estimates for PM_2.5_ on non-wildfire days in models controlling for minimum, mean, and maximum temperature were 1.003 (CI: 0.984–1.022), 1.007 (CI: 0.988–1.026), 1.013 (CI: 0.995–1.032), respectively; while the lag 0 estimates for the increase in the association of PM due to the presence of wildfire smoke were 6.6% (CI: 2.5–10.8%), 6.1% (CI: 2.1–10.3%), 5.1% (CI: 1.2–9.2%), respectively. No meaningful differences in the primary results were found when a seven-day rolling average of daily mean RH was included in the model in addition to daily mean temperature (Tables S1, S4 and Figs. 3, S4), nor were there meaningful differences in the results of the two-fraction model in any of the sensitivity analyses (Table S5 and Figs. 4, S5-S7).

## Discussion

We found that the presence of wildfire smoke increases the association of PM_2.5_ and PM_10_ with asthma visits at shorter lags (lag 0 and lags 0–2). We did not find a significant increase in association for PM_10–2.5_ at any lag, nor did we find a significant increase in association at lags 0–6 for any PM fraction.

The similarity between estimates for PM_2.5_ and PM_10_ when wildfire smoke was present was probably due to the high correlation (0.96) between PM_2.5_ and PM_10_ on wildfire days, which suggests that a change in wildfire PM_10_ was roughly equivalent to a change in wildfire PM_2.5_. It is possible that we did not see a significant increase in association at lags 0–6 for any PM fraction because there were two few wildfire days preceded by six other consecutive wildfire days—only 50 days, compared to 107 days for lags 0–2 and 175 days for lag 0. This may have made our sample size too small. Similarly, we may not have detected a significant association of PM_10–2.5_ with asthma visits on wildfire days because the variation of PM_10–2.5_ on wildfire days (SD: 4.8) was much lower than that of PM_2.5_ (SD: 14.8) and PM_10_ (SD: 16.5). It is worth noting that the lower CIs for wildfire PM_10–2.5_ at lag 0 and lags 0–2 were quite close to excluding a rate ratio of 1.0, suggesting that a larger sample size might be able to detect a significant association for PM_10–2.5_ on wildfire days (and possibly an increase in association compared to non-wildfire days).

Our study used models very similar to those of Deflorio-Barker et al. [12], Reid et al. [13], and Delfino et al. [14], which also included an interaction of PM with an indicator for days affected by wildfire smoke. However, a key difference in our study is that we excluded wildfire days where PM_2.5_ concentrations exceeded the maximum PM_2.5_ concentration on non-wildfire days. We believe that this is important, since the association of wildfire PM with asthma may be stronger at higher concentrations of PM than at lower concentrations [3, 13]. By eliminating wildfire days with excessively high PM_2.5_ concentrations (and, by extension, excessively high PM_10_ concentrations), we ensured that the stronger associations we found for wildfire PM compared to non-wildfire PM could not be attributed to a nonlinear association between PM and asthma visits.

Despite removing wildfire days with extremely high PM_2.5_ concentrations, our results were similar to those of Deflorio-Barker et al. [12], Reid et al. [13], and Delfino et al. [14], who all found that PM_2.5_ had a stronger association with asthma visits or hospitalizations during wildfire periods than during non-wildfire periods. We thus showed that the stronger associations of wildfire PM with asthma visits found in these studies cannot be solely attributed to differences in the concentration of wildfire PM and non-wildfire PM. Furthermore, since there were mixed results from these studies regarding whether there was a statistically significant increase in the association of PM_2.5_ with asthma during wildfires, our study added further support to the hypothesis that the observed increases were not due to random variation. Our results were especially meaningful since we contrasted the associations of non-wildfire PM and wildfire PM at relatively low concentrations (i.e., at or below 40.4 μg/m^3^ PM_2.5_ for lag 0 analyses), when the association of PM with asthma visits would be expected to be less clear. We also examined a relatively long time period of 6 years that included 175 days where the air quality was affected by wildfire smoke. In contrast, the study by DeFlorio-Barker et al. [12] examined 3 years, while Reid et al. [13] and Delfino et al. [14] examined single wildfire smoke events. However, the study by DeFlorio-Barker et al. covered the United States, rather than just one city, and it is likely that their estimates represented the effects of PM on asthma patients over a much broader range of wildfire fuelbed types and burn conditions than our study.

During our study period, air quality in Reno was affected by major wildfires from the Klamath, Northern Coast, and Sierra Nevada mountain ranges. Using BlueSky Playground v3.0 (https://tools.airfire.org/playground/v3/emissionsinputs.php), we determined that the top five Fuel Characteristic Classification System fuelbed types of these wildfires were mixed Jeffery pine forest, mixed Douglas fir forest, red fir forest, oak woodlands, and chamise chaparral shrublands. Since fuelbed types are unique to every region, our results may not be generalizable to regions where fuelbed types are very different, such as the Southeastern United States.

The mechanisms which could make wildfire PM more or less harmful than non-wildfire PM for patients with asthma are likely to be complex, but there are at least three broad categories into which these mechanisms may fall: (1) differences in the composition of wildfire and non-wildfire PM, (2) interactions between PM and temperature, and (3) patient behavioral changes. These mechanistic categories are not mutually exclusive, and it is possible that each of them may play an important role in the harmfulness of wildfire PM.

Perhaps the best supported mechanism for the increased harmfulness of wildfire PM is a change in PM composition during wildfire events. The effects of wildfire PM on respiratory tissue has been compared with that of non-wildfire PM in several laboratory studies. Nakayama Wong et al. [9] and Verma et al. [10] showed that cell-culture response to PM from wildfires differed from that of non-wildfire PM, though it was not clear in either of these studies whether one was more harmful than the other [23]. However, other studies have suggested that PM from wildfires is much more harmful than non-wildfire PM. In a study using live mice, Wegesser et al. [11] estimated that PM_2.5_ and PM_10–2.5_ from wildfires was about 10 times as toxic as an equivalent mass of non-wildfire PM. Similarly, Franzi et al. [8] conducted an in vitro study on PM from the same wildfire as Wegesser et al. and found that coarse PM (defined as PM between 10.2–2.1 μm in diameter) from the wildfire was approximately four times as toxic as non-wildfire coarse PM. It is not clear whether the results of Wegesser et al. and Franzi et al. are generalizable to all wildfires, since Kim et al. [24] reported that the toxicity of PM from wildfires depends on the activity status of the wildfire (flaming or smoldering) and the fuel type. Thus, more research is needed to determine the conditions under which PM from wildfires is the most toxic, but it at least seems that wildfire PM is more toxic than non-wildfire PM under some conditions.

A second possible mechanism for an increased PM association with adverse asthma events during wildfires is a PM-temperature interaction, since wildfire events tend to be associated with higher ambient temperatures [25]. Shaposhnikov et al. [26] found a statistically significant interaction between temperature and PM_10_ during a combined heat-wave/wildfire event in Moscow, Russia. Other studies have reported that temperature modifies the effect of non-wildfire PM as well [27–30]. Part of the modifying effect of temperature may be mediated through changes in PM composition, so it is not possible to make a clear causal distinction between the two mechanisms. However, temperature could also modify the effect of PM on patients with asthma by inducing behavioral changes, since more people are likely to be outside for longer periods of time during the warm season than during the cold season [31]. This possibility deserves further investigation, as it could explain a large proportion of the increased risk due to wildfire PM_2.5_ compared to non-wildfire PM_2.5_ that we observed in our study.

However, it is also possible that patients with asthma change their behavior in other ways that would ameliorate the harmful effects of wildfire smoke, such as increasing their use of rescue medications. Lipner et al. [32] observed that lung function in children aged 12–21 years initially decreased following a smoke exposure event (at a 1 day lag) but subsequently increased to above normal (at a 2 day lag). They speculated that this was due to older children effectively managing their symptoms using rescue medications. Another study found that prescriptions fills for rescue medications increased during smoke events [33]. Both of these studies support the notion that patients with asthma are likely to take proactive action during a wildfire smoke event that reduces the risk of an ED or UC visit. Since our study uses ED and UC visits as health endpoints, such behavior could cause wildfire PM to appear less harmful in relation to PM from other sources.

Other than the composition of wildfire PM, possible PM-temperature interactions, and changes in patient behavior, the harmfulness of wildfire smoke may also be affected by particle size. In general, there is stronger evidence for the harmfulness of PM_2.5_ than PM_10–2.5_ [34], regardless of whether or not the source is a wildfire. However, studies comparing the toxicity of wildfire PM_2.5_ and PM_10–2.5_ have produced mixed results [23]. Leonard et al. [35] concluded that ultrafine particles were the most reactive (and hence more harmful), while Jalava et al. [36] found that the toxicity of the different size fractions depended on how toxicity was measured. Dong et al. [23] attributed the differences between these two studies to differences in the distance the PM was transported by the wind before it was collected. Wegesser et al. [11] found equal toxicity for both PM_2.5_ and PM_10–2.5_, but noted that real-world exposure mechanisms might produce different levels of toxicity in practice. The results of our study could be construed as support for the hypothesis that finer wildfire PM is more hazardous for patients with asthma. However, the lack of a statistically significant positive modification of PM_10–2.5_ by wildfires in our study may also be attributed to the very small increase in mean PM_10–2.5_ levels on wildfire days compared to non-wildfire days (Table 1), which suggests that most of the PM_10–2.5_ observed on wildfire days is coming from non-wildfire sources. Thus, in addition to more research on PM composition, more research is needed on the relative toxicity of different size fractions of PM.

It may also be worthwhile to investigate the role that the PM_2.5_/PM_10–2.5_ ratio plays in the harmfulness of wildfire smoke, since the ratio differs between wildfire days and non-wildfire days (Figs. 2 and 4). This possibility does not appear to have been investigated yet. Future work could also focus on differences in the health effects of smoke from a prescribed burn versus a wildfire. In our study, no distinction was made between prescribed burns and wildfires, but differences in the relative toxicity of the smoke produced could have important implications for forest management policy.

Our study had several limitations. The most important of these was not being able to directly distinguish between PM from wildfires and PM from other sources. Since we instead compared the association of PM with asthma visits on days affected by wildfire smoke and days not affected, our study likely underestimated the difference between the health effects of wildfire PM and non-wildfire PM. We were also limited by the necessity of dropping extremely high PM_2.5_ wildfire days in order to make an appropriate comparison with non-wildfire days. Other than during the intermittent wildfire smoke event, levels of air pollution were relatively low in Reno, so a study like ours would perhaps be more effective in a city with higher levels of non-wildfire PM. A third limitation, which applies to most ecological studies like ours, was not being able to assess individual exposure levels. Although we combined data from four different air monitors throughout Reno, exposures may vary a great deal depending on where individuals live and work, and depending on the amount of time they spend outdoors.

A key strength of our study was that it spanned 6 years and included large and small wildfire events, in contrast to many studies which examined a single large wildfire event. Thus, our data included wildfires that occurred under a variety of conditions and at a wide range of distances from Reno, making our estimates robust to variability between fires.

## Conclusions

The main conclusion of this study was that PM from wildfires can be more harmful for patients with asthma than PM from other sources, as we found statistically significant increases in the association of PM_2.5_ and PM_10_ with emergency visits for asthma on wildfire days versus non-wildfire days. This validated previous studies which found similar results but were not able to consistently detect a statistically significant difference. Another important contribution of our study was that we showed that the stronger association of wildfire PM with asthma visits cannot be solely attributed to higher concentrations of PM on wildfire days versus non-wildfire days, since the effects of wildfire and non-wildfire PM were compared at similar concentrations, and these concentrations were relatively low compared to the concentrations that often occur during a wildfire smoke event. Thus, there appears to be factors other than increased PM concentration that contribute to the increased harmfulness of wildfire smoke. More research is needed to understand these factors, but current research suggests that the harmfulness of wildfire PM may be affected by fuel source and burn rate [24], ambient temperature [26], behavioral changes [31–33], and smoke transport distance [23].

## Supplementary information

**Additional file 1.**

## Data Availability

The datasets used and/or analyzed during the current study are available from the corresponding author on reasonable request. In addition, air quality data is publicly available from the EPA’s internet database at https://www.epa.gov/airdata [37].

## Abbreviations

ACF: Autocorrelation function
CI: Confidence interval
ED: Emergency department
EHR: Electronic health records
EPA: Environmental Protection Agency
GAM: Generalized additive model
HYSPLIT: Hybrid Single Particle Lagrangian Integrated Trajectory
IRB: Institutional Review Board
PM: Particulate matter
PM_2.5_: Fine particulate matter (0–2.5 μm in diameter)
PM_10_: Particulate matter (0–10 μm in diameter)
PM_10–2.5_: Coarse particulate matter (2.5–10 μm in diameter)
RH: Relative humidity
SD: Standard deviation
UC: Urgent care
UNR: University of Nevada, Reno
WCHD-AQMD: Washoe County Health District Air Quality Management Division
