# Supplementary material for: Particulate matter and emergency visits for asthma: a time-series study of their association in the presence and absence of wildfire smoke in Reno, Nevada, 2013–2018
Source: Environ Health. 2020 Aug 27;19:92. doi: 10.1186/s12940-020-00646-2 (PMC7453527; doi:10.1186/s12940-020-00646-2)
Supplement: Supplementary file 1 — Additional file 1. [file 12940_2020_646_MOESM1_ESM.docx]

**Supplemental Material**

**Particulate matter and emergency visits for asthma: comparison of their associations in the presence and absence of wildfire smoke in Reno, Nevada, 2013-2018**

Daniel Kiser, William J. Metcalf, Gai Elhanan, Brendan Schnieder, Karen Schlauch, Andrew Joros, Craig Petersen, Joseph Grzymski

**Table of Contents**

Table S1 – Rate ratios for the change in asthma visits due to a 5 µg/m^3^ increase in PM on non-wildfire and wildfire days in the single-fraction models (primary analysis).

Table S2 – Rate ratios for the change in asthma visits due to a 5 µg/m^3^ increase in PM on non-wildfire and wildfire days in the single-fraction models (sensitivity analysis with a rolling seven-day average of daily minimum temperature substituted for a rolling seven-day average of daily mean temperature in the base model).

Table S3 – Rate ratios for the change in asthma visits due to a 5 µg/m^3^ increase in PM on non-wildfire and wildfire days in the single-fraction models (sensitivity analysis with a rolling seven-day average of daily maximum temperature substituted for a rolling seven-day average of daily mean temperature in the base model).

Table S4 – Rate ratios for the change in asthma visits due to a 5 µg/m^3^ increase in PM on non-wildfire and wildfire days in the single-fraction models (sensitivity analysis with a rolling seven-day average of daily mean relative humidity included in the base model in addition to a rolling seven-day average of daily mean temperature).

Table S5 – Estimated coefficients from the two-fraction model: comparison between the primary and sensitivity analyses.

Figure S1 – Histogram of daily hours of recorded wildfire smoke exposure for the 188 wildfire days designated in this study.

Figure S2 – Rate ratios the effect of PM on for asthma visits to the ED and UC centers in the presence or absence of wildfire smoke (sensitivity analysis with a rolling seven-day average of daily minimum temperature substituted for a rolling seven-day average of daily mean temperature in the base model). PM was scaled to 5 µg/m^3^ for all fractions and lags. Numeric labels between points are estimates of the change in the rate ratio when wildfire smoke is present compared to when it is not present, presented as a factor. This change can also be presented as a percent (i.e., the factor 1.028 is equivalent to an increase of 2.8%), which is how the changes are presented in the body of the paper. Rate ratios and the differences between them are presented in tabular form in Table S2.

Figure S3 – Rate ratios for the effect of PM on asthma visits to the ED and UC centers in the presence or absence of wildfire smoke (sensitivity analysis with a rolling seven-day average of daily maximum temperature substituted for a rolling seven-day average of daily mean temperature in the base model). PM was scaled to 5 µg/m^3^ for all fractions and lags. Numeric labels between points are estimates of the change in the rate ratio when wildfire smoke is present compared to when it is not present, presented as a factor. This change can also be presented as a percent (i.e., the factor 1.028 is equivalent to an increase of 2.8%), which is how the changes are presented in the body of the paper. Rate ratios and the differences between them are presented in tabular form in Table S3.

Figure S4 – Rate ratios for the effect of PM on asthma visits to the ED and UC centers in the presence or absence of wildfire smoke (sensitivity analysis with a rolling seven-day average of daily mean relative humidity included in the base model in addition to a rolling seven-day average of daily mean temperature). PM was scaled to 5 µg/m^3^ for all fractions and lags. Numeric labels between points are estimates of the change in the rate ratio when wildfire smoke is present compared to when it is not present, presented as a factor. This change can also be presented as a percent (i.e., the factor 1.028 is equivalent to an increase of 2.8%), which is how the changes are presented in the body of the paper. Rate ratios and the differences between them are presented in tabular form in Table S4.

Figure S5 – Percent increase in asthma visits due to wildfire smoke, at given levels of PM_2.5_ and PM_10-2.5_ (sensitivity analysis with a rolling seven-day average of daily minimum temperature substituted for a rolling seven-day average of daily mean temperature in the base model). Estimates were constrained to the regions within 5 µg/m^3^ of a wildfire data point to limit extrapolation. Shaded regions indicate estimates that were significant at the 0.05 level.

Figure S6 – Percent increase in asthma visits due to wildfire smoke, at given levels of PM_2.5_ and PM_10-2.5_ (sensitivity analysis with a rolling seven-day average of daily maximum temperature substituted for a rolling seven-day average of daily mean temperature in the base model). Estimates were constrained to the regions within 5 µg/m^3^ of a wildfire data point to limit extrapolation. Shaded regions indicate estimates that were significant at the 0.05 level.

Figure S7 – Percent increase in asthma visits due to wildfire smoke, at given levels of PM_2.5_ and PM_10-2.5_ (sensitivity analysis with a rolling seven-day average of daily mean relative humidity included in the base model in addition to a rolling seven-day average of daily mean temperature). Estimates were constrained to the regions within 5 µg/m^3^ of a wildfire data point to limit extrapolation. Shaded regions indicate estimates that were significant at the 0.05 level.

Table S1 – Rate ratios for the change in asthma visits due to a 5 µg/m^3^ increase in PM on non-wildfire and wildfire days (primary analysis).

|  |  | Rate Ratios | |  |  |  |  |
| --- | --- | --- | --- | --- | --- | --- | --- |
| PM | Non-wildfire days | | Wildfire days | | $\Delta$ | | |
| PM_2.5_ |  | |  | |  | | |
| Lag 0 | 1.007 (0.988, 1.026) | | 1.068 (1.033, 1.105)* | | 1.061 (1.021, 1.103)* | | |
| Lags 0-2 | 1.014 (0.992, 1.037) | | 1.083 (1.031, 1.138)* | | 1.068 (1.012, 1.127)* | | |
| Lags 0-6 | 1.019 (0.992, 1.046) | | 1.088 (1.015, 1.167)* | | 1.068 (0.992, 1.150) | | |
| PM_10-2.5_ |  | |  | |  | | |
| Lag 0 | 1.010 (0.998, 1.023) | | 1.057 (0.998, 1.119) | | 1.046 (0.987, 1.109) | | |
| Lags 0-2 | 1.016 (1.001, 1.031)* | | 1.100 (0.994, 1.218) | | 1.083 (0.978, 1.200) | | |
| Lags 0-6 | 1.026 (1.007, 1.045)* | | 0.902 (0.691, 1.176) | | 0.879 (0.674, 1.146) | | |
| PM_10_ |  | |  | |  | | |
| Lag 0 | 1.006 (0.998, 1.015) | | 1.062 (1.032, 1.092)* | | 1.055 (1.025, 1.086)* | | |
| Lags 0-2 | 1.009 (0.999, 1.019) | | 1.082 (1.036, 1.130)* | | 1.072 (1.026, 1.120)* | | |
| Lags 0-6 | 1.013 (1.001, 1.025)* | | 1.082 (1.009, 1.160)* | | 1.068 (0.995, 1.146) | | |

Note: $\Delta$: multiplicative change in the rate ratio when wildfire smoke is present versus when wildfire smoke is not present, given by $\exp\left( \beta_{2} \right)$ (see equation 1). 95% confidence intervals for each estimate are included in parentheses.

* Confidence intervals that do not span 1.0.

Table S2 – Rate ratios for the change in asthma visits due to a 5 µg/m^3^ increase in PM on non-wildfire and wildfire days (sensitivity analysis with a rolling seven-day average of daily minimum temperature substituted for a rolling seven-day average of daily mean temperature in the base model).

|  |  | Rate Ratios | |  |  |  |  |
| --- | --- | --- | --- | --- | --- | --- | --- |
| PM | Non-wildfire days | | Wildfire days | | $\Delta$ | | |
| PM_2.5_ |  | |  | |  | | |
| Lag 0 | 1.003 (0.984, 1.022) | | 1.069 (1.033, 1.106)* | | 1.066 (1.025, 1.108)* | | |
| Lags 0-2 | 1.009 (0.986, 1.032) | | 1.082 (1.030, 1.137)* | | 1.073 (1.016, 1.133)* | | |
| Lags 0-6 | 1.011 (0.984, 1.039) | | 1.085 (1.012, 1.163)* | | 1.073 (0.997, 1.156) | | |
| PM_10-2.5_ |  | |  | |  | | |
| Lag 0 | 1.006 (0.993, 1.019) | | 1.049 (0.990, 1.111) | | 1.043 (0.984, 1.105) | | |
| Lags 0-2 | 1.009 (0.993, 1.025) | | 1.087 (0.982, 1.203) | | 1.077 (0.973, 1.193) | | |
| Lags 0-6 | 1.017 (0.998, 1.037) | | 0.895 (0.686, 1.167) | | 0.879 (0.674, 1.147) | | |
| PM_10_ |  | |  | |  | | |
| Lag 0 | 1.004 (0.995, 1.012) | | 1.060 (1.030, 1.090)* | | 1.056 (1.026, 1.087)* | | |
| Lags 0-2 | 1.006 (0.995, 1.016) | | 1.078 (1.033, 1.126)* | | 1.072 (1.026, 1.121)* | | |
| Lags 0-6 | 1.008 (0.996, 1.021) | | 1.078 (1.005, 1.156)* | | 1.069 (0.996, 1.147) | | |

Note: $\Delta$: multiplicative change in the rate ratio when wildfire smoke is present versus when wildfire smoke is not present, given by $\exp\left( \beta_{2} \right)$ (see equation 1). 95% confidence intervals for each estimate are included in parentheses.

* Confidence intervals that do not span 1.0.

Table S3 – Rate ratios for the change in asthma visits due to a 5 µg/m^3^ increase in PM on non-wildfire and wildfire days (sensitivity analysis with a rolling seven-day average of daily maximum temperature substituted for a rolling seven-day average of daily mean temperature in the base model).

|  |  | Rate Ratios | |  |  |  |  |
| --- | --- | --- | --- | --- | --- | --- | --- |
| PM | Non-wildfire days | | Wildfire days | | $\Delta$ | | |
| PM_2.5_ |  | |  | |  | | |
| Lag 0 | 1.013 (0.995, 1.032) | | 1.065 (1.030, 1.102)* | | 1.051 (1.012, 1.092)* | | |
| Lags 0-2 | 1.023 (1.001, 1.045)* | | 1.080 (1.028, 1.135)* | | 1.056 (1.001, 1.114)* | | |
| Lags 0-6 | 1.028 (0.999, 1.059) | | 1.099 (1.026, 1.178)* | | 1.069 (0.992, 1.152) | | |
| PM_10-2.5_ |  | |  | |  | | |
| Lag 0 | 1.015 (1.003, 1.028)* | | 1.062 (1.003, 1.125)* | | 1.046 (0.987, 1.109) | | |
| Lags 0-2 | 1.023 (1.008, 1.039)* | | 1.110 (1.002, 1.229)* | | 1.084 (0.979, 1.202) | | |
| Lags 0-6 | 1.036 (1.018, 1.055)* | | 0.907 (0.695, 1.184) | | 0.875 (0.671, 1.142) | | |
| PM_10_ |  | |  | |  | | |
| Lag 0 | 1.009 (1.001, 1.018)* | | 1.062 (1.032, 1.093)* | | 1.052 (1.022, 1.083)* | | |
| Lags 0-2 | 1.014 (1.004, 1.024)* | | 1.082 (1.036, 1.130)* | | 1.067 (1.021, 1.115)* | | |
| Lags 0-6 | 1.019 (1.008, 1.031)* | | 1.080 (1.007, 1.158)* | | 1.059 (0.987, 1.137) | | |

Note: $\Delta$: multiplicative change in the rate ratio when wildfire smoke is present versus when wildfire smoke is not present, given by $\exp\left( \beta_{2} \right)$ (see equation 1). 95% confidence intervals for each estimate are included in parentheses.

* Confidence intervals that do not span 1.0.

Table S4 – Rate ratios for the change in asthma visits due to a 5 µg/m^3^ increase in PM on non-wildfire and wildfire days (sensitivity analysis with a rolling seven-day average of daily mean relative humidity included in the base model in addition to a rolling seven-day average of daily mean temperature).

|  |  | Rate Ratios | |  |  |  |  |
| --- | --- | --- | --- | --- | --- | --- | --- |
| PM | Non-wildfire days | | Wildfire days | | $\Delta$ | | |
| PM_2.5_ |  | |  | |  | | |
| Lag 0 | 1.008 (0.989, 1.027) | | 1.068 (1.032, 1.105)* | | 1.059 (1.019, 1.101)* | | |
| Lags 0-2 | 1.015 (0.993, 1.038) | | 1.082 (1.030, 1.137)* | | 1.066 (1.010, 1.126)* | | |
| Lags 0-6 | 1.020 (0.993, 1.047) | | 1.087 (1.014, 1.166)* | | 1.066 (0.990, 1.149) | | |
| PM_10-2.5_ |  | |  | |  | | |
| Lag 0 | 1.010 (0.997, 1.023) | | 1.053 (0.994, 1.116) | | 1.043 (0.984, 1.106) | | |
| Lags 0-2 | 1.015 (1.000, 1.031) | | 1.098 (0.991, 1.216) | | 1.081 (0.976, 1.198) | | |
| Lags 0-6 | 1.026 (1.007, 1.045)* | | 0.901 (0.691, 1.176) | | 0.879 (0.674, 1.146) | | |
| PM_10_ |  | |  | |  | | |
| Lag 0 | 1.006 (0.998, 1.015) | | 1.061 (1.031-1.091)* | | 1.054 (1.024, 1.085)* | | |
| Lags 0-2 | 1.009 (0.999, 1.019) | | 1.081 (1.035, 1.129)* | | 1.071 (1.025, 1.119)* | | |
| Lags 0-6 | 1.013 (1.001, 1.025)* | | 1.081 (1.007, 1.159)* | | 1.066 (0.994, 1.145) | | |

Note: $\Delta$: multiplicative change in the rate ratio when wildfire smoke is present versus when wildfire smoke is not present, given by $\exp\left( \beta_{2} \right)$ (see equation 1). 95% confidence intervals for each estimate are included in parentheses.

* Confidence intervals that do not span 1.0.

Table S5 – Estimated coefficients from the two-fraction model: comparison between the primary and sensitivity analyses.

|  | Coefficients | | |  | Estimate 1 | |  |  | Estimate 2 | |  |
| --- | --- | --- | --- | --- | --- | --- | --- | --- | --- | --- | --- |
| Analyses | $\hat{\beta_{2}}$ | $\hat{\beta_{4}}$ | $\hat{\beta_{5}}$ | % | | *p*-value | | % | | *p*-value | |
| Primary | 0.0747 | 0.0341 | - 0.2259 | 19.1 | | 0.00092 | | 32.8 | | 0.00022 | |
| Sensitivity |  |  |  |  | |  | |  | |  | |
| Min Temp | 0.0718 | 0.0326 | - 0.2163 | 18.4 | | 0.00126 | | 31.4 | | 0.00034 | |
| Max Temp | 0.0720 | 0.0350 | - 0.2308 | 17.6 | | 0.00213 | | 30.9 | | 0.00047 | |
| Humidity | 0.0722 | 0.0334 | - 0.2192 | 18.5 | | 0.00148 | | 31.7 | | 0.00041 | |

Note: Min Temp: rolling average of daily mean temperature replaced by rolling average of daily minimum temperature; Max Temp: rolling average of daily mean temperature replaced by rolling average of daily maximum temperature; Humidity: rolling average of daily mean relative humidity included in addition to rolling average of daily mean temperature; $\left( \exp\left( \hat{\beta_{2}}PM_{2.5}+ \hat{\beta_{4}}PM_{10-2.5}+ \hat{\beta_{5}} \right)-1 \right)\times100$: equation used to estimate percent increase in asthma visits due to the presence of wildfire smoke (equation 3, $PM_{2.5}$ and $PM_{10-2.5}$ scaled to 5 µg/m^3^); Estimate 1: PM_2.5_ at 20 µg/m^3^ and PM_10-2.5_ at 15 µg/m^3^; Estimate 2: PM_2.5_ at 25 µg/m^3^ and PM_10-2.5_ at 20 µg/m^3^.

Figure S1 – Histogram of daily hours of recorded wildfire smoke exposure for the 188 wildfire days designated in this study.


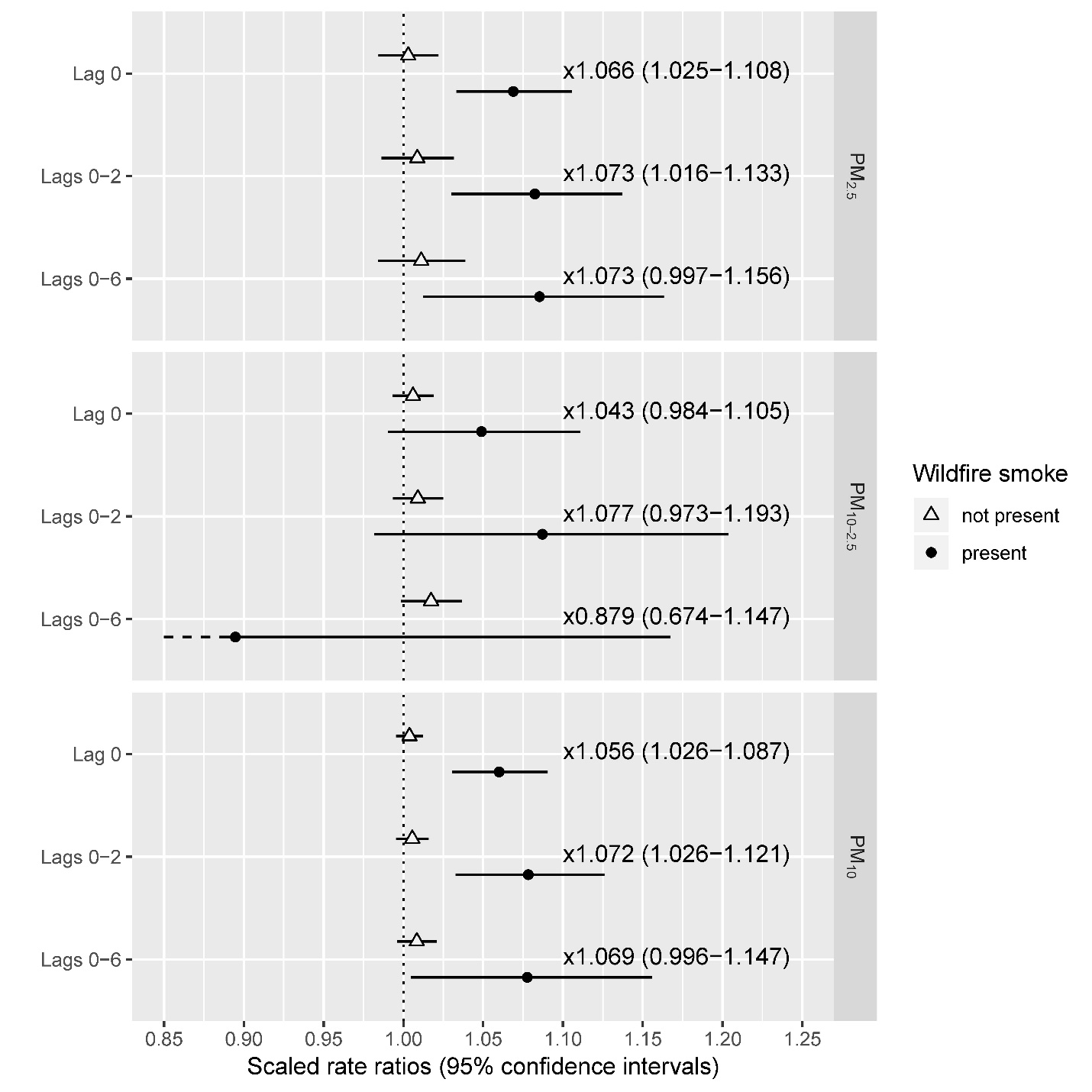


Figure S2 – Rate ratios for the effect of PM on asthma visits to the ED and UC centers in the presence or absence of wildfire smoke (sensitivity analysis with a rolling seven-day average of daily minimum temperature substituted for a rolling seven-day average of daily mean temperature in the base model). PM was scaled to 5 µg/m^3^ for all fractions and lags. Numeric labels between points are estimates of the change in the rate ratio when wildfire smoke is present compared to when it is not present, presented as a factor. This change can also be presented as a percent (i.e., the factor 1.028 is equivalent to an increase of 2.8%), which is how the changes are presented in the body of the paper. Rate ratios and the differences between them are presented in tabular form in Table S2.


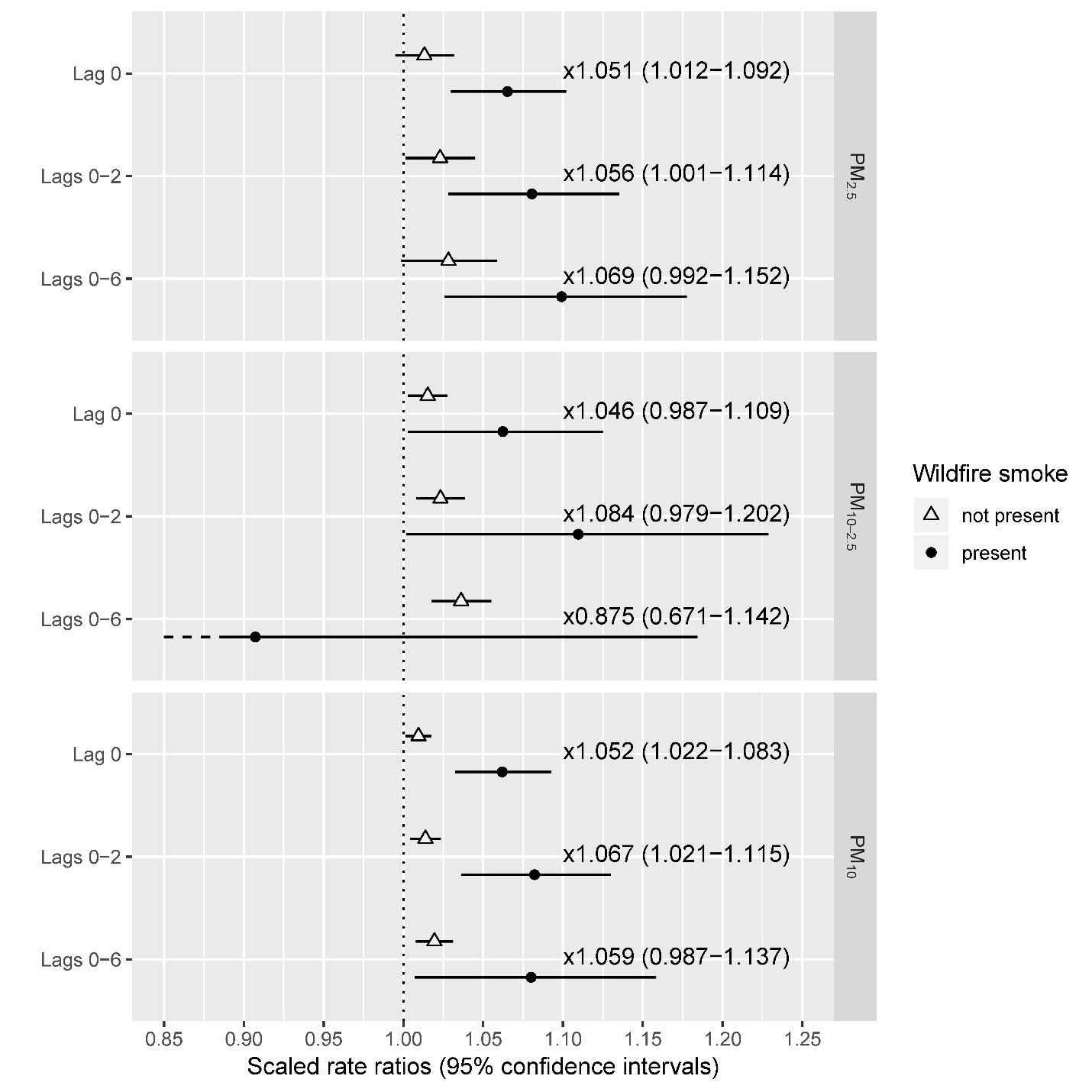


Figure S3 – Rate ratios for the effect of PM on asthma visits to the ED and UC centers in the presence or absence of wildfire smoke (sensitivity analysis with a rolling seven-day average of daily maximum temperature substituted for a rolling seven-day average of daily mean temperature in the base model). PM was scaled to 5 µg/m^3^ for all fractions and lags. Numeric labels between points are estimates of the change in the rate ratio when wildfire smoke is present compared to when it is not present, presented as a factor. This change can also be presented as a percent (i.e., the factor 1.028 is equivalent to an increase of 2.8%), which is how the changes are presented in the body of the paper. Rate ratios and the differences between them are presented in tabular form in Table S3.


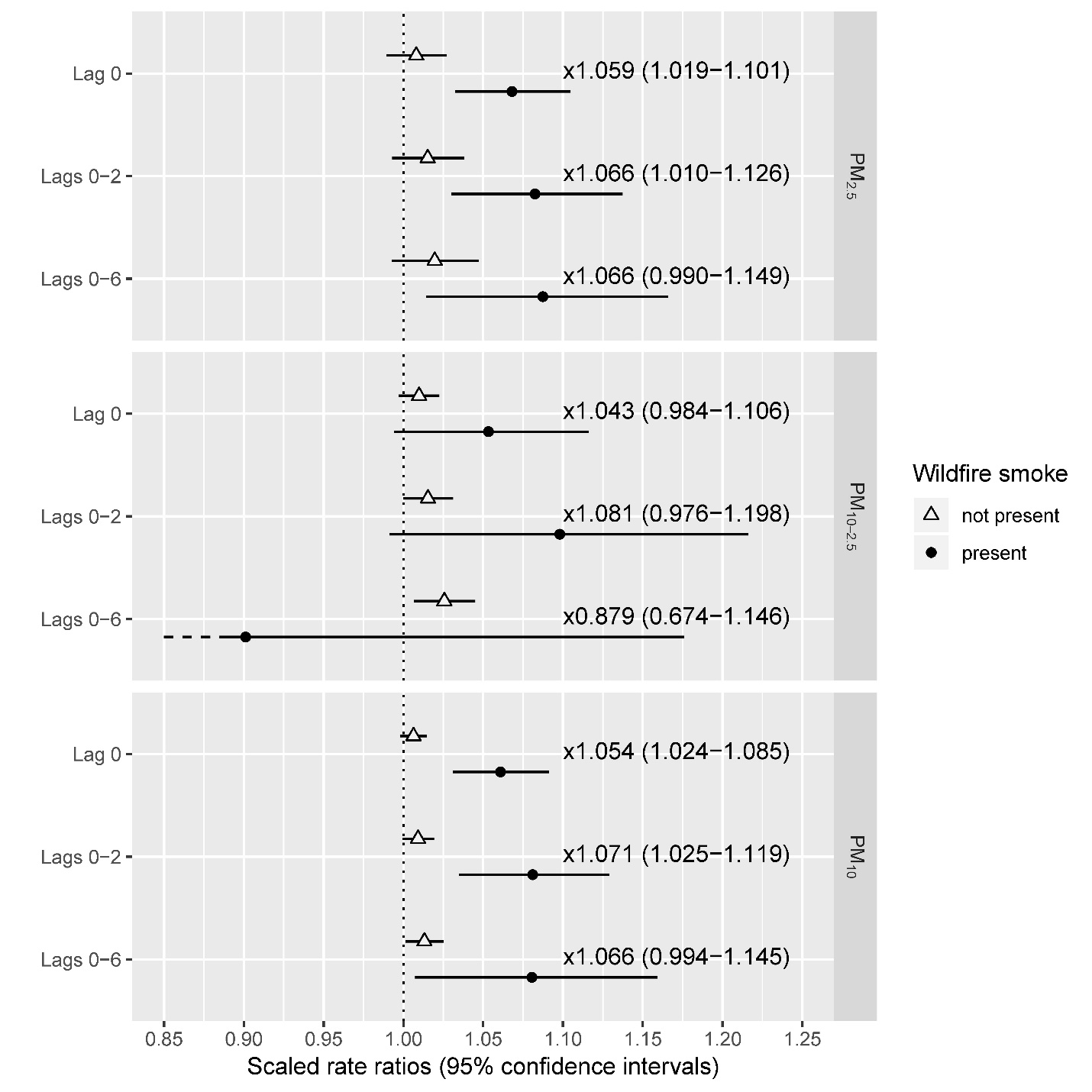


Figure S4 – Rate ratios for the effect of PM on asthma visits to the ED and UC centers in the presence or absence of wildfire smoke (sensitivity analysis with a rolling seven-day average of daily mean relative humidity included in the base model in addition to a rolling seven-day average of daily mean temperature). PM was scaled to 5 µg/m^3^ for all fractions and lags. Numeric labels between points are estimates of the change in the rate ratio when wildfire smoke is present compared to when it is not present, presented as a factor. This change can also be presented as a percent (i.e., the factor 1.028 is equivalent to an increase of 2.8%), which is how the changes are presented in the body of the paper. Rate ratios and the differences between them are presented in tabular form in Table S4.


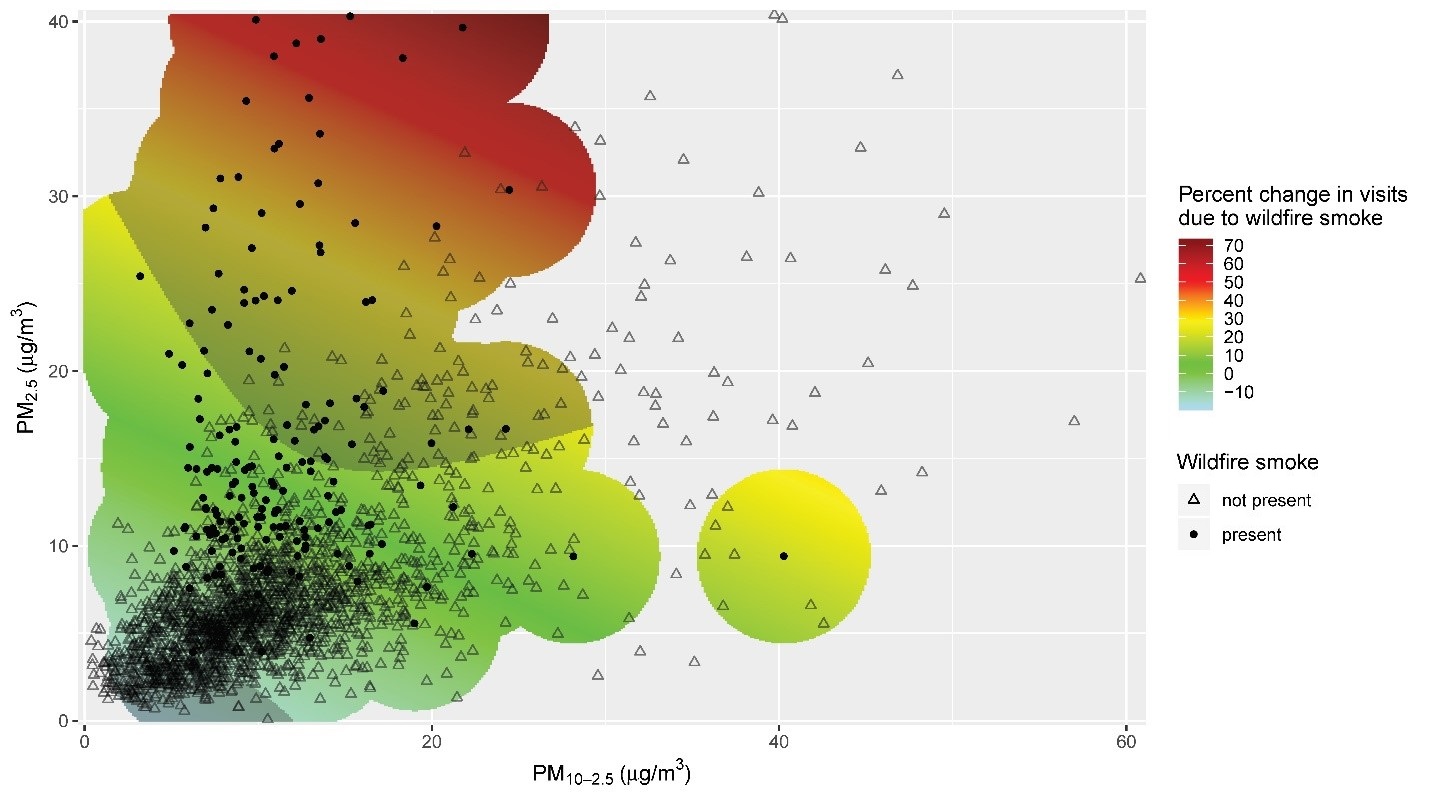


Figure S5 – Percent increase in asthma visits due to wildfire smoke, at given levels of PM_2.5_ and PM_10-2.5_ (sensitivity analysis with a rolling seven-day average of daily minimum temperature substituted for a rolling seven-day average of daily mean temperature in the base model). Estimates were constrained to the regions within 5 µg/m^3^ of a wildfire data point to limit extrapolation. Shaded regions indicate estimates that were significant at the 0.05 level.


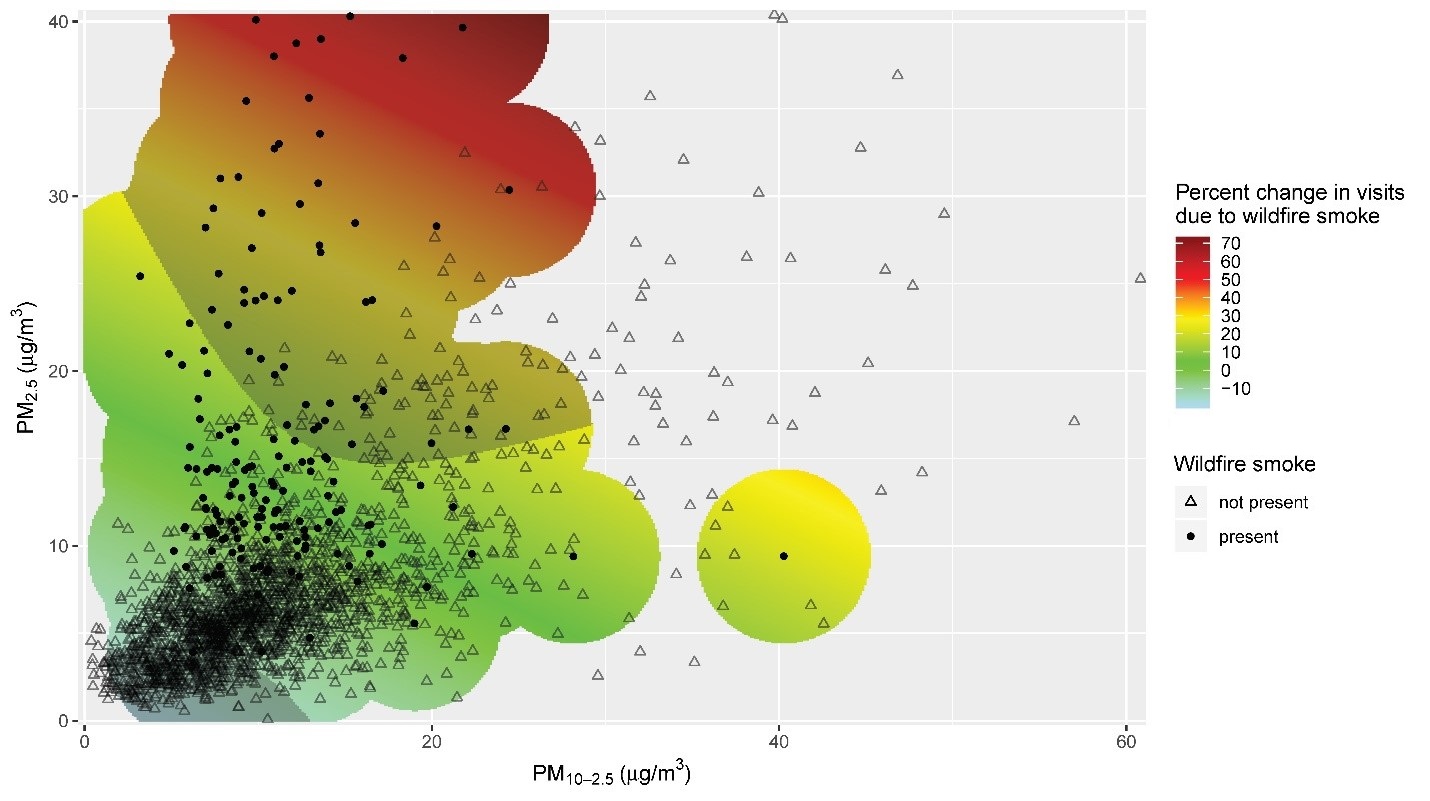


Figure S6 – Percent increase in asthma visits due to wildfire smoke, at given levels of PM_2.5_ and PM_10-2.5_ (sensitivity analysis with a rolling seven-day average of daily maximum temperature substituted for a rolling seven-day average of daily mean temperature in the base model). Estimates were constrained to the regions within 5 µg/m^3^ of a wildfire data point to limit extrapolation. Shaded regions indicate estimates that were significant at the 0.05 level.


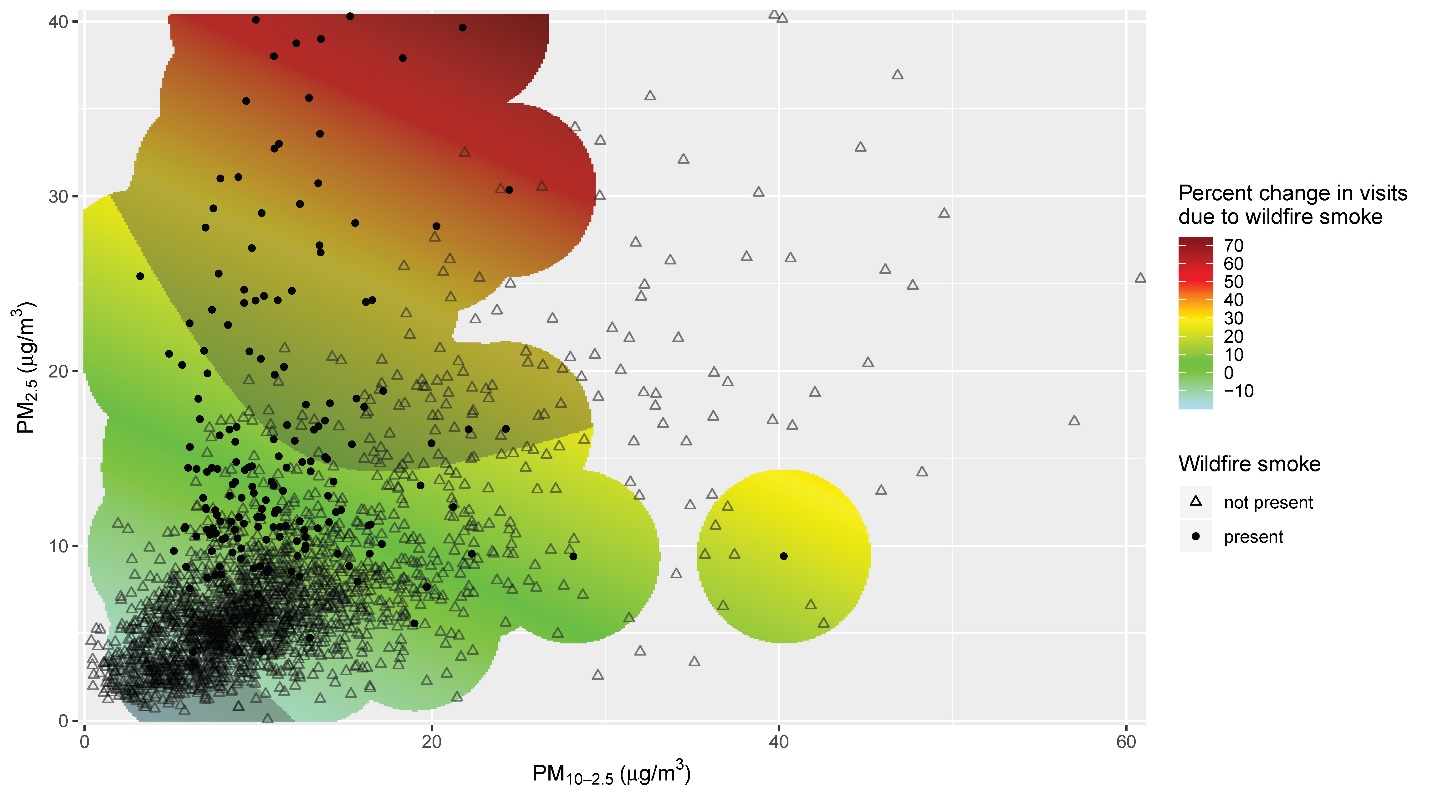


Figure S7 – Percent increase in asthma visits due to wildfire smoke, at given levels of PM_2.5_ and PM_10-2.5_ (sensitivity analysis with a rolling seven-day average of daily mean relative humidity included in the base model in addition to a rolling seven-day average of daily mean temperature). Estimates were constrained to the regions within 5 µg/m^3^ of a wildfire data point to limit extrapolation. Shaded regions indicate estimates that were significant at the 0.05 level.
